# Editorial: Breakthroughs in immunotherapies and precision treatment against infectious diseases

**DOI:** 10.3389/fcimb.2022.953312

**Published:** 2022-08-03

**Authors:** Ting Huang, Jing Zhang, Roberta Antonia Diotti, Qinqin Pu

**Affiliations:** ^1^ Antibiotics Research and Re-evaluation Key Laboratory of Sichuan Province, School of pharmacy, Chengdu University, Chengdu, China; ^2^ Department of Systems Biology, The University of Texas MD Anderson Cancer Center, Houston, TX, United States; ^3^ Laboratory of Medical Microbiology and Virology, “Vita-Salute” San Raffaele University, Milan, Italy; ^4^ Department of Microbiology, University of Pennsylvania, Pennsylvania, PA, United States

**Keywords:** immunotherapies, SARS-Cov-2, bacteria, fungi, parasite

A variety of new and reemerging pathogenic organisms, including bacteria, viruses, parasites, and fungi, have a great influence on public health worldwide. Novel immunotherapies and precision treatments are considered as potent approaches in treatment of the infectious diseases caused by these pathogenic organisms. Immunotherapy is a specific type of biological therapy that employs substances to activate or suppress the immune system of the host to recognize and eliminate these pathogenic organisms for fighting infectious diseases. It consists of several types of white blood cells, organs, and tissues of the immune system. Some immunotherapies target specific cells of the host immune system, such as immune inhibitors (Matteucci et al.) and monoclonal antibodies (Song et al.). Other immunotherapies alter the immune system in a general way, including various vaccines and immune system modulators (Matteucci et al.). Precision treatments, sometimes known as “precision medicine,” are a pioneering method to tailoring disease prevention and treatments of different patients (Zhan et al.). This state-of-the-art approach considers the differences in an individual patient’s special physical condition (Zhang et al. and Mo et al.), environments, and lifestyles.

Although great efforts have been made to treat severe infectious diseases in the past few decades, the currently available therapies or treatments are insufficient or unsatisfactory against the pathogens. Therefore, there is an urgent need to develop new therapies for improving efficacy and fighting infectious disease. The aim of this Research Topic is to present the novel therapeutic strategies that are accessible to combat the pathogens mentioned above. Our Research Topic brings together six interesting articles that summarize the main knowledge on the breakthroughs in immunotherapies and precision treatments against some severe and commensal infectious diseases, such as COVID-19, mycosis, tuberculosis, and malaria.

The first article of this Research Topic is a mini review paper. In this review, Song et al. provide a comprehensive overview on the origins, structures, pathogenesis, transmission, and variation of SARS-CoV-2, which is a pathogen that is causing the COVID-19 pandemic worldwide. In addition, they describe the clinical characteristics and current strategies to treat (monoclonal antibodies and drugs based on oligonucleotide) and prevent COVID-19 and highlight the problems and challenges in the long run. Then, Matteucci et al. discuss the potential targets for treatment or control of two severe infectious diseases, tuberculosis (TB) and malaria, based on current evidence in regard to the interactions between various hosts and pathogens. Notably, they present an important overview of diverse approaches, including immune inhibitors (such as Zileuton, an anti-inflammatory drug for TB) that have been tested in experimental models or clinical trials and that will accelerate development of potent treatments for combating both TB and malaria.

Next, Zhan et al. found that pulmonary mycosis by mixed fungal infections frequently occurs in patients who are not obviously immunocompromised in a case series report. The surgery they propose is a good choice to treat patients with a poor response in some mixed pulmonary mycoses. They suggest that it is better to identify the cause of illness by a rigorous procedure to make a precision treatment plan before performing systemic antifungal therapy. Allogeneic hematopoietic stem cell transplantation (allo-HSCT) is a common treatment for patients with acute leukemia (AL). But there is a deficiency in the treatment: the patients are often infected by cytomegalovirus (CMV) after allo-HSCT. In an interesting study, Shen et al. successfully establish a compendious model to predict the intractable CMV infection after HSCT. Importantly, this model presents an appropriate time window for prevention of endangered CMV infections in patients with HID HSCT. Thus, it will greatly help to improve the current allo-HSCT curative therapy. In regard to the CMV infection, two papers from the Zhang et al. and Mo et al. groups further provide an important description of the retrospective studies of severe aplastic anemia patients receiving either pre-HSCT or post-HSCT caused by CMV. They found that both pretransplant platelet refractoriness and alternative donors were considered as remarkable targets to predict cytomegalovirus retinitis (CMVR) in patients with aplastic anemia. Additionally, dynamic detecting of T lymphocyte subsets and serum immunoglobulin levels of post-HSCT might contribute to examining a program of CMVR in HSCT patients with aplastic anemia. Those interesting studies will provide new insights into precision therapy for patients facing special medical conditions or complex syndromes.

In conclusion, this Research Topic brings together these intriguing articles that provide a global overview of the current understanding regarding the breakthroughs in immunotherapies and precision treatments against the severe and commensal infectious diseases mentioned above. The editors of this topic highly appreciate all authors and reviewers for their valuable contributions to this collection.

## Author contributions

All authors listed have made a substantial, direct, and intellectual contribution to the work and approved it for publication.

## Funding

TH is supported by the National Natural Science Foundation of China (No. 31900120).

## Acknowledgments

We would like to thank all the authors and reviewers who have participated in this Research Topic for their valuable contributions. We also thank Dr. Chen Li (Free University of Berlin, Germany) for his help in this Research Topic.

## Conflict of interest

The authors declare that the research was conducted in the absence of any commercial or financial relationships that could be construed as a potential conflict of interest.

## Publisher’s note

All claims expressed in this article are solely those of the authors and do not necessarily represent those of their affiliated organizations, or those of the publisher, the editors and the reviewers. Any product that may be evaluated in this article, or claim that may be made by its manufacturer, is not guaranteed or endorsed by the publisher.

